# Enhancing osteogenesis and angiogenesis functions for Ti-24Nb-4Zr-8Sn scaffolds with methacrylated gelatin and deferoxamine

**DOI:** 10.3389/fbioe.2024.1372636

**Published:** 2024-04-19

**Authors:** Qian Xu, Yun Bai, Shujun Li, Wentao Hou, Yulin Hao, Rui Yang, Xiaowu Li, Xing Zhang

**Affiliations:** ^1^ Department of Materials Physics and Chemistry, School of Materials Science and Engineering, Key Laboratory for Anisotropy and Texture of Materials, Ministry of Education, Northeastern University, Shenyang, Liaoning, China; ^2^ Institute of Metal Research, Chinese Academy of Sciences, Shenyang, Liaoning, China; ^3^ School of Materials Science and Engineering, University of Science and Technology of China, Hefei, Anhui, China

**Keywords:** Ti2448, GelMA, bone scaffolds, deferoxamine, angiogenesis

## Abstract

Repair of large bone defects remains challenge for orthopedic clinical treatment. Porous titanium alloys have been widely fabricated by the additive manufacturing, which possess the elastic modulus close to that of human cortical bone, good osteoconductivity and osteointegration. However, insufficient bone regeneration and vascularization inside the porous titanium scaffolds severely limit their capability for repair of large-size bone defects. Therefore, it is crucially important to improve the osteogenic function and vascularization of the titanium scaffolds. Herein, methacrylated gelatin (GelMA) were incorporated with the porous Ti-24Nb-4Zr-8Sn (Ti2448) scaffolds prepared by the electron beam melting (EBM) method (Ti2448-GelMA). Besides, the deferoxamine (DFO) as an angiogenic agent was doped into the Ti2448-GelMA scaffold (Ti2448-GelMA/DFO), in order to promote vascularization. The results indicate that GelMA can fully infiltrate into the pores of Ti2448 scaffolds with porous cross-linked network (average pore size: 120.2 ± 25.1 μm). Ti2448-GelMA scaffolds facilitated the differentiation of MC3T3-E1 cells by promoting the ALP expression and mineralization, with the amount of calcium contents ∼2.5 times at day 14, compared with the Ti2448 scaffolds. Impressively, the number of vascular meshes for the Ti2448-GelMA/DFO group (∼7.2/mm^2^) was significantly higher than the control group (∼5.3/mm^2^) after cultivation for 9 h, demonstrating the excellent angiogenesis ability. The Ti2448-GelMA/DFO scaffolds also exhibited sustained release of DFO, with a cumulative release of 82.3% after 28 days. Therefore, Ti2448-GelMA/DFO scaffolds likely provide a new strategy to improve the osteogenesis and angiogenesis for repair of large bone defects.

## 1 Introduction

The large bone defect repair remains challenging for orthopedic clinical treatment (Vidal et al., 2020; Zhang et al., 2023). With the advances of 3D printing technology, porous titanium alloys have been widely developed as bone scaffolds, due to good osteoconductivity and osteointegration (Niinomi et al., 2016; Chen et al., 2023; Yuan et al., 2023). Previous studies have found that 3D printed Ti-24Nb-4Zr-8Sn (Ti2448) showed the good biocompatibility and low elastic modulus, close to that of the human bone (Nune et al., 2017a; Tang et al., 2021). Nevertheless, the bare scaffolds lack the bioactive components that are critical for stimulating osteogenesis (Jahr et al., 2021). Moreover, the growth of blood vessels inside the bone scaffold remains great challenge for large bone defect repair. (Koons et al., 2020). Thus, the development of porous titanium alloys with improved bioactivity is of particular interest.

The vascularization is crucially important for repair of large bone defects (Wang et al., 2018). After the bone fracture, the inflammation and hematoma are immediately established, following by the formation of blood clots at ∼1–5 days (Yang and Xiao, 2020). During the soft callus formation at ∼5–16 days, the endothelial cells from the blood vessels provide angiocrine factors such as BMP-2, Noggin and IL-33 to the osteoprogenitor cells or mesenchymal stem cells (MSCs), leading to the cell differentiation to osteoblasts and further ossification (Chen et al., 2020; Salhotra et al., 2020). During the hard callus formation at ∼16–21 days, osteoblasts can secrete angiogenic factors such as VEGF and FGF, which act on endothelial cells and further promote the vascular growth (Raines et al., 2019; Hendriks and Ramasamy, 2020; Chen et al., 2021). During the remodeling stage at ∼21–35 days, blood vessels can also participate in clearing the bone metabolites and improve the repair effects. Thus, blood vessel formation and bone regeneration occur in a coupled manner (Liu and Castillo, 2018; Ha et al., 2022). Sufficient blood vessels distributed in distance ∼100–300 μm are critical to ensure the sufficient supply of oxygen and nutrients, as well as removal of waste products (Marrella et al., 2018). While the number of blood vessels is not enough, cell death, inadequate and delayed blood circulation may occur, ultimately causing inner bone tissue necrosis (Kang et al., 2016; Liu et al., 2017; Marrella et al., 2018). For example, the 3D-printed porous Ti6Al4V scaffolds were implanted in the longitudinal axis of rabbit radius (1.5 cm in length) (Ma et al., 2021). Post surgery for 12 weeks, only a few newly formed bone tissues were found inside the porous Ti6Al4V scaffolds with sparse blood vessels (Ma et al., 2021). The collagen modified titanium-based implants induced angiogenic activity via *in vitro* tubule formation as compared to bared titanium-based implants, but no differences were noticed in angiogenesis and osteointegration *in vivo.* However, by incorporating the vascular endothelial growth factors (VEGF) into the collagen modified titanium-based implants, both bone growth and vascular regeneration were significantly improved (Li J et al., 2021). Therefore, achieving effective vascular regeneration during the repair of large bone defects remains a great challenge.

DFO is a commonly used drug, which can activate the HIF-1α pathway by chelating Fe^3+^, thereby promoting the expression of downstream signaling molecule VEGF, and facilitating vascular regeneration (Ran et al., 2018; Cheng et al., 2020; Xue et al., 2020). However, DFO can be easily filtered and cleared by kidneys, resulting in a relatively short drug residence time in the human body (t_1/2_ = 5 min, in mice) (Park et al., 2022). Therefore, suitable carriers are necessitate to sustained release of DFO with extended life time. For example, DFO-gelatin microspheres loaded with type I collagen and fibronectin were prepared and dispersed in 10 mL PBS at 37°C, which showed a long sustained release of DFO up to 20 days (Zeng et al., 2022). Herein, the biocompatible methacryloyl group-grafted gelatin denoted as GelMA has been selected for DFO loading, which can be incorporated into the porous Ti2448 scaffolds, which may promote internal vascularization and further bone regeneration.

In this study, 3D printed Ti2448 scaffolds with low elastic modulus have been selected as the loading-bearing part (Liu et al., 2016; Li et al., 2019). Ti2448-GelMA scaffolds are further prepared. DFO is then doped into the Ti2448-GelMA scaffold as the angiogenic agent. The chemical composition and micro-structures of Ti2448-GelMA and Ti2448-GelMA/DFO scaffolds are characterized. The proliferation, adhesion, and differentiation of MC3T3-E1 cells cultured on these scaffolds are evaluated. Additionally, the angiogenic effects of DFO are investigated by culturing human umbilical vascular endothelial cells (HUVECs) on the Ti2448-GelMA/DFO scaffolds. The release of DFO from the Ti2448-GelMA/DFO scaffolds has also been investigated. The Ti2448-GelMA/DFO scaffolds as prepared can obviously enhance the osteogenic differentiation ability and vascular regeneration, demonstrating great potential application or large bone defect repair.

## 2 Experimental procedure

### 2.1 The synthesis and characterization of GelMA

Type A porcine skin gelatin (Sigma, St. Louis, USA) was fully dissolved in phosphate buffer saline (PBS) solution at 50°C (10% w/v). The methacrylic anhydride (MAA, 8% v/v) was then added and stirred at 200 rpm for 24 h. Dulbecco’s phosphate-buffered saline (DPBS) was preheated to 50°C under the same volume with PBS. The preheated DPBS was then mixed with the above mixed solution at 50°C for 10 min. The final solution was dialyzed against distilled water at 40°C for 5 days, using a dialysis membrane with 12–14 kDa cutoff (Fisher Scientific, Waltham, USA), then lyophilized for 5 days to yield the purified gelatin methacrylate (GelMA).

### 2.2 The fabrication of Ti2448-GelMA and Ti2448-GelMA/DFO scaffolds

The 3D-printed porous Ti2448 scaffolds were prepared by an Arcam A1 EBM system (Arcam, Gothenburg, Sweden), following the same method as previously reported (Xu et al., 2022). In brief, the scaffold models (9.50 mm × 9.50 mm × 2.50 mm) with a nominal 70% porosity were created. The Ti2448 powders (particle size ∼45–106 µm) were preheated to 773 K before melting. The samples were produced with a scan speed of 130 mm/s, a voltage of 60 kV, and a vacuum of 2 × 10^−3^ mbar.

The lithium phenyl-2,4,6-trimethylbenzoylphosphinate (LAP) photoinitiator (0.1% w/v) was added to the GelMA solution in PBS (5% w/v), and then filtered through 0.22 µm membrane filter. 100 μL GelMA solution was added to the Ti2448 scaffolds, which was exposed to the UV light (365 nm) both top and bottom sides for 2 min. The samples were then lyophilized for 24 h, denoted as Ti2448-GelMA. The DFO powder was added to the above 5% w/v GelMA solution, with the final DFO concentration of 0.5 wt%, and LAP was then added. Following the same procedure as Ti2448-GelMA preparation, Ti2448-GelMA scaffolds loaded with DFO are acquired and denoted as Ti2448-GelMA/DFO.

### 2.3 The characterization of GelMA and Ti2448-GelMA scaffolds

The chemical structure of gelatin and GelMA was characterized by ^1^H nuclear magnetic resonance spectrometer (^1^H-NMR) with a Bruker Avance Neo 400 spectrometer (Bruker, Bremen, Germany) using D_2_O as the solvent.

Fourier transform infrared spectrometry (FTIR, Agilent Technologies, California, USA) was conducted in the range of 400–4,000 cm^−1^. The crystal information was measured by X-ray diffraction (Rigaku, Tokyo, Japan) using the radiation of Cu Kα radiation (λ = 1.5418 Å). The surface morphology and elemental analysis was investigated by a scanning electron microscope (SEM, Carl Zeiss AG, Jena, Germany) equipped with energy dispersive spectroscopy (EDS, Oxford Instruments, Abingdon, UK). The mean pore size of GelMA network was evaluated by the ImageJ software based on SEM images.

### 2.4 Cell viability and proliferation

MC3T3-E1 cells were purchased from the Cell Bank of the Chinese Academy of Sciences (Shanghai, China), and incubated in the α-MEM medium (Hyclone, GE Healthcare, Chicago, USA) supplemented with 10% FBS (Lonsera, Uruguay) and 1% antibiotic/antimycotic (Gen-View Scientific Inc., Calimesa, USA). MC3T3-E1 cells (3 × 10^4^ cells/scaffolds) were seeded on both Ti2448 and Ti2448-GelMA scaffolds in a 48-well plate for 1, 3, and 5 days. The cytotoxicity was evaluated by the CCK-8 reagent (Gen-View Scientific Inc., Calimesa, USA). Briefly, the 100 μL mixture (10 μL CCK-8 and 90 μL culture medium) was added to each well for 3 h and the OD value was measured with a microplate reader (Multiskan Go, Thermo Fisher Scientific, Waltham, USA) under 450 nm.

MC3T3-E1 cells (3 × 10^4^ cells/scaffolds) were seeded on Ti2448, Ti2448-GelMA and Ti2448-GelMA/DFO scaffolds. After culturing for 24 h, cells on the scaffold were gently rinsed by PBS, and immobilized in 4% paraformaldehyde for 2 h, following by dehydration in a graded series of ethanol solutions (50%, 70%, 90%, 95% and 100%) for 10 min each and dried with hexamethyldisilane for 20 min. The morphology was observed by SEM.

### 2.5 Osteogenic differentiation of MC3T3-E1 cells

Alkaline phosphatase (ALP) activities of MC3T3-E1 cells cultured on both Ti2448 and Ti2448-GelMA scaffolds were evaluated. MC3T3-E1 cells were seeded on the scaffolds (3 × 10^4^ cells/scaffold) for 7 and 14 days, which were lysed in 100 μL of Triton X-100 (1% v/v) for 5 min. The ALP activity was determined by ALP quantification kit (Beyotime, Shanghai, China) and the OD value was measured at 520 nm.

Alizarin Red S (ARS) staining was used to evaluate the biomineralization level. After cultured for 7 and 14 days, cells were fixed with 4% paraformaldehyde for 15 min. The fixed samples were stained with ARS solution (Beijing Solarbio Science and Technology Co., Ltd., Beijing, China) for 30 min and cleaned with PBS. Finally, the cells are observed utilizing a confocal microscope (Olympus Corporation, Tokyo, Japan). For quantification analysis, 10% hexadecyl pyridinium chloride monohydrate (Sigma-Aldrich, St. Louis, USA) was used to dissolve the mineralized nodules and the OD value was measured at 562 nm.

### 2.6 *In vitro* tube formation experiment

*In vitro* angiogenesis of Ti2448-GelMA and Ti2448-GelMA/DFO scaffolds was investigated by the tube formation assay. The pipette tips, 96-well plates and Matrigel matrix (ABW, Shanghai, China) were first placed at 4°C overnight. 50 μL Matrigel matrix were then added to the plates for 30 min Ti2448-GelMA and Ti2448-GelMA/DFO scaffolds were first immersed into 2 mL serum-free α-MEM at 100 rpm for 24 h. The serum-free α-MEM was incorporated into the extracts to generate a range of diluted extracts (1/2, 1/4 and 1/8 concentrations). The serially diluted extraction medium was prepared by supplemention with 10% FBS and 1% antibiotic/antimycotic, hereafter referred to as 1, 1/2, and 1/4 dilution group. Cells (3 × 10^4^ cells/scaffold) with different diluted extraction were seeded on the Matrigel coated plate for 3, 6 and 9 h. The morphology of HUVEC cells were observed by an Eclipse Ti inverted fluorescent microscope (Nikon Instruments Inc., Tokyo, Japan). Quantitative analysis for the number of vascular meshes were calculated by ImageJ software.

The release of DFO from the Ti2448-GelMA/DFO scaffolds was evaluated by a mould incubator (Shanghai Longyue Instrument Equipment Co., Ltd, Shanghai, China). The Ti2448-GelMA/DFO scaffolds were first incubated in the centrifuge tube at 37°C under 100% humidity. After 1, 3, 7 and 14 days, the scaffolds were removed and rinsed with deionized water. Then more deionized water was collected and supplied in the centrifuge tube until the final volume is 2 mL. The obtained solution was then chelated with FeCl_3_ (10 mg/mL) at a 9:1 volumetric ratio for 5 min. The OD value was measured at 485 nm. The amount of DFO released was calculated according to the standard DFO calibration curve. The drug release rate is calculated using the amount of released drug divided by the total drug content.

### 2.7 Statistical analysis

All data were expressed as means ± standard deviation. One-way analysis of variance (ANOVA) was conducted for multiple samples with varying time points. Statistical significance was determined by *p* < 0.05.

## 3 Results and discussion

### 3.1 The characterization of GelMA

GelMA was first fabricated by the reaction between the MAA and the functional groups of lysine and hydroxyl lysine in gelatin (Supplementary Figure S1A). GelMA was further photo crosslinked to form three-dimensional network structures. The ^1^H spectra of gelatin and GelMA were conducted to confirm the synthesis of GelMA (Supplementary Figure S1B). Two new peaks were found at 5.45 ppm and 5.68 ppm, corresponding to vinyl from the methacrylate group. However, both peaks were not present in the gelatin sample. The peak at 2.81 ppm represented the methylene proton (2H) of lysine, which showed high intensity peak of the unmodified gelatin and low intensity peak of the GelMA, consistent with previous findings (Cao et al., 2021; He et al., 2023). Supplementary Figure S2 showed that GelMA as prepared was amorphous. FTIR results (Supplementary Figure S1C) revealed the characteristic peaks of gelatin and GelMA. The peak at 1,543 cm^-1^ from the amide bond was related to N-H bending. Furthermore, the peak at 1,641 cm^-1^ for gelatin was associated with the C=O stretching of amide bond, while that for GelMA associated with both C=O and C=C stretching exhibited higher intensity, indicating the presence of methacrylate groups in the GelMA sample. The presence of these characteristic peaks again confirmed the successful synthesis of GelMA.

### 3.2 Preparation and characterization of Ti2448-GelMA scaffolds

**FIGURE 1:**
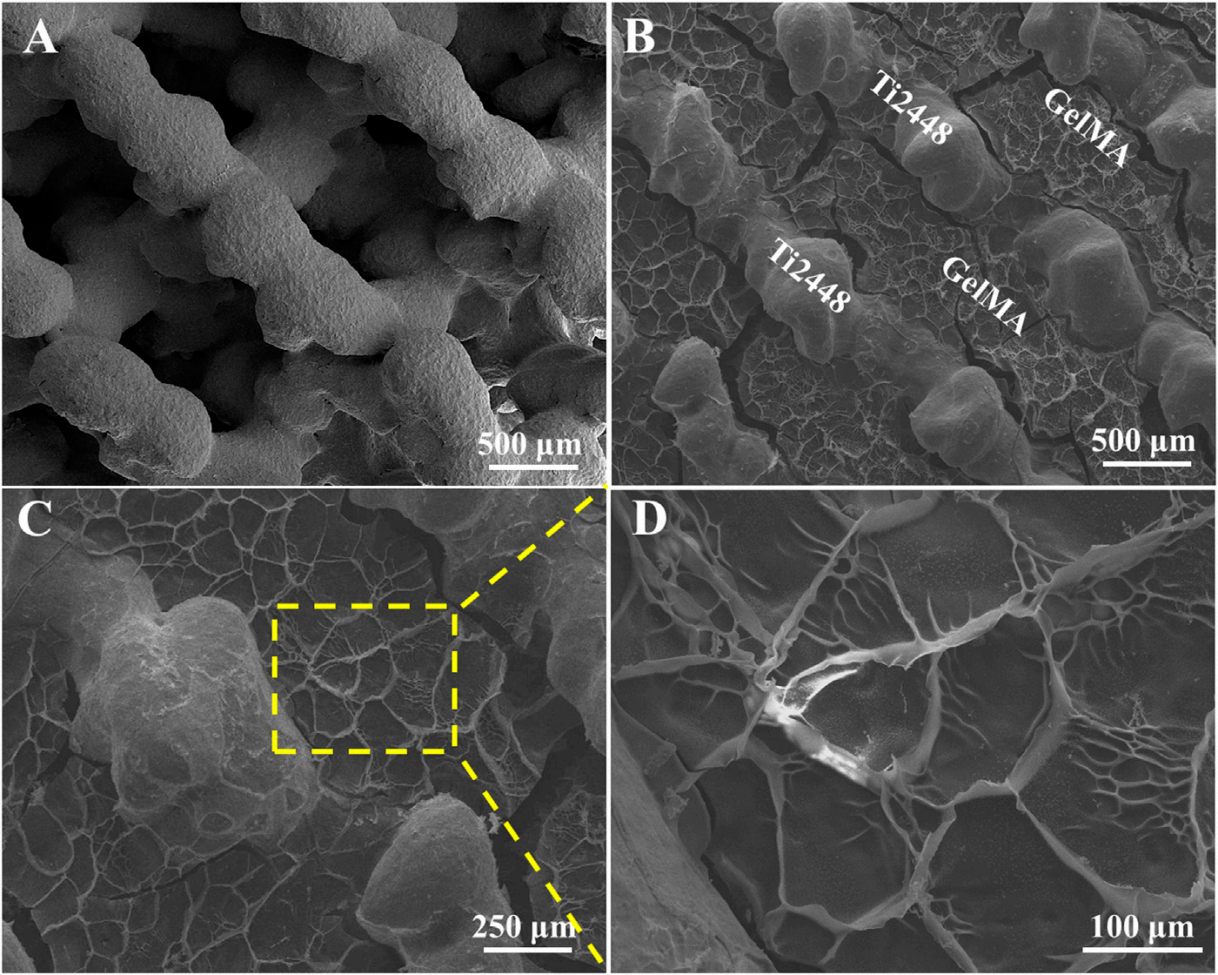
The morphology of **(A)** Ti2448 and **(B–D)** Ti2448-GelMA scaffolds.

Previous study reported that Ti2448 have good biocompatibility and osteoconductivity (Nune et al., 2017b; Liu et al., 2019). Ti2448 scaffolds showed the porous structure with the pore size of ∼600 μm (Figure 1A). GelMA, mimicking the extracellular matrix, offered a three-dimensional structure and support the osteogenic differentiation of bone marrow mesenchymal stem cells, leading to further bone regeneration (Piao et al., 2021). For Ti2448-GelMA scaffolds, GelMA was incorporated into the porous interconnected structure of Ti2448 scaffolds (Figure 1B) and formed a porous network with the mean pore size of 120.2 ± 25.1 μm (Figures 1C,D). The interconnectivity and proper pore size (>100 μm) were crucially important for the transportation of oxygen/nutrients, cell attachment as well as the vascular growth, which were beneficial for the bone regeneration (Cimenoglu et al., 2011; Wu et al., 2014; Safaei-Yaraziz et al., 2021; Uiiah et al., 2021; Yao et al., 2021). Taking the advantages of 3D printing technology, the porous Ti2448-GelMA scaffolds provides the good interconnectivity with proper pore size (120.2 ± 25.1 μm), which may enhance the vessel growth and further facilitate the bone regeneration.

**FIGURE 2:**
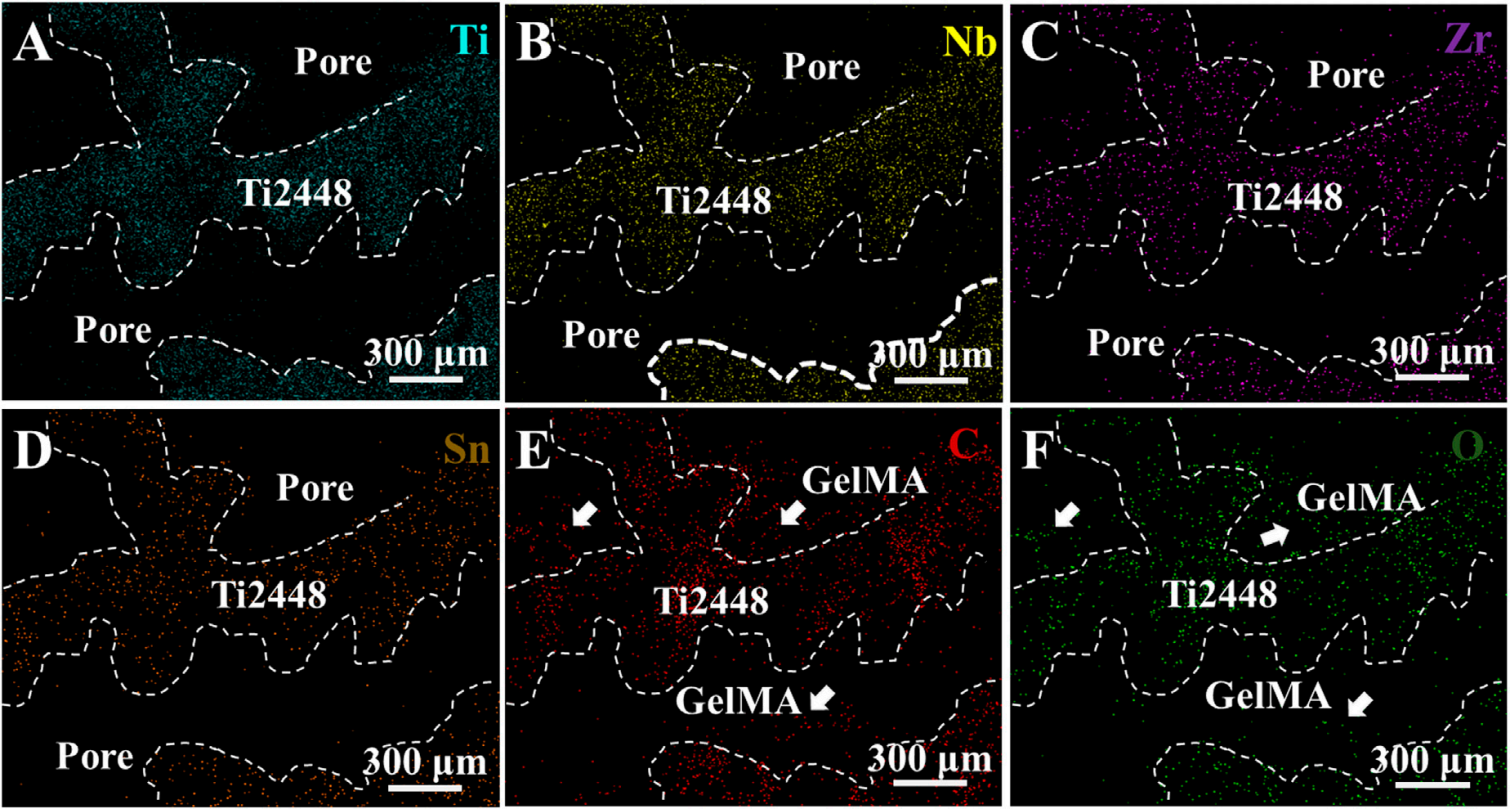
EDS mapping of Ti2448-GelMA scaffolds for **(A)** Ti, **(B)** Nb, **(C)** Zr, **(D)** Sn, **(E)** C and **(F)** O elements.

EDS mapping of Ti, Nb, Zr, Sn, C, and O elements in the Ti2448-GelMA scaffold was conducted (Figure 2). The presence of C and O elements further proved the incorporation of GelMA into the Ti2448 scaffolds. Overall, GelMA was successfully loaded into the Ti2448 scaffold.

### 3.3 *In vitro* cytocompatibility of Ti2448-GelMA scaffolds

**FIGURE 3:**
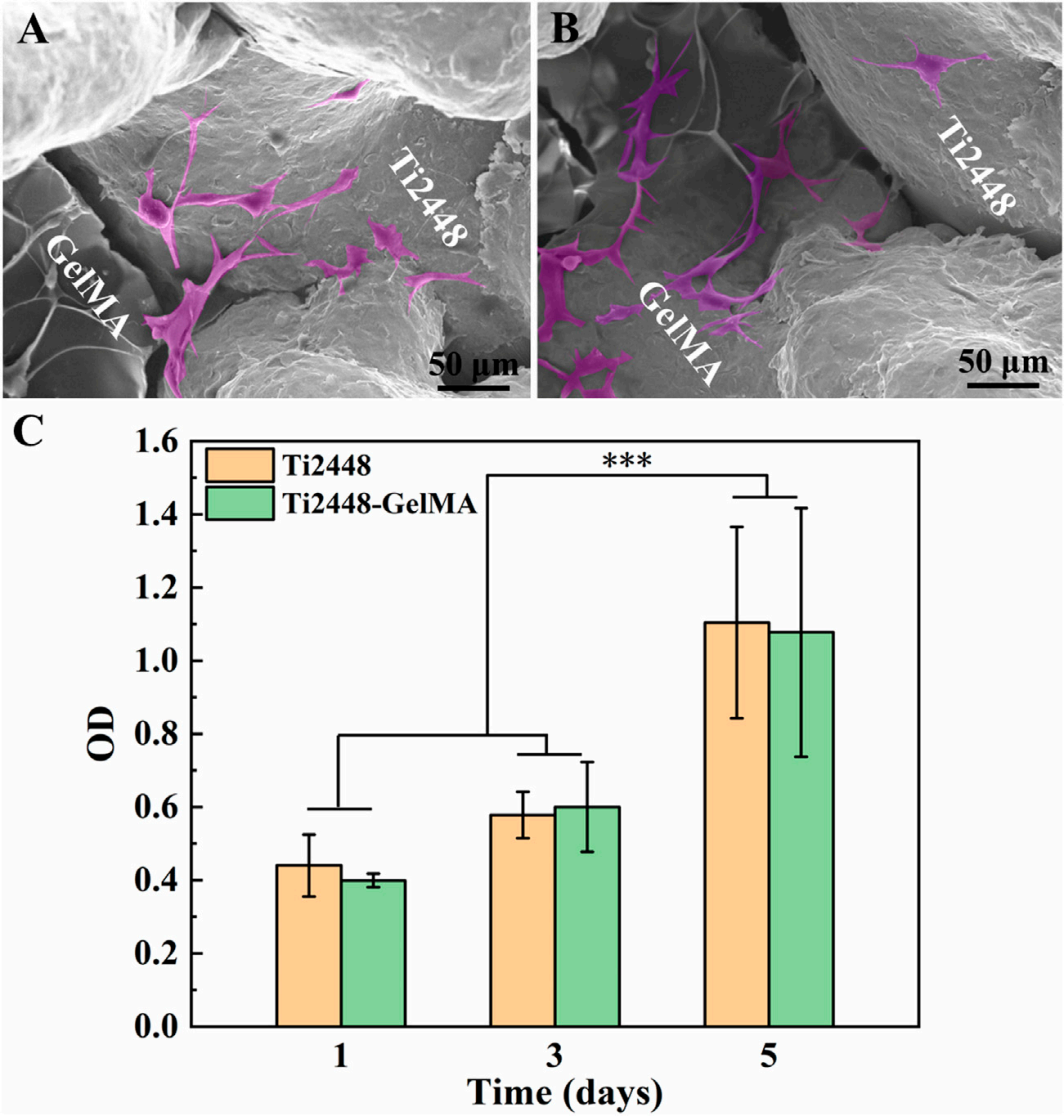
**(A, B)** Cell morphology of MC3T3-E1 cells on Ti2448-GelMA scaffold for 1 day, **(C)** CCK-8 assay of MC3T3-E1 cells on Ti2448 and Ti2448-GelMA scaffold surfaces for 1, 3, and 5 days. Cells were highlighted with a pink color based on the gray values of the images. (***p < 0.001)

The morphology of MC3T3-E1 cells cultured on Ti2448-GelMA scaffolds was evaluated after 1 day culture (Figures 3A, B). Cells spread well with the pseudopodia extending outwards on both Ti2448 and GelMA surfaces. Notably, the cells exhibited unhindered adhesion and traversal across the Ti2448 scaffold and GelMA surfaces simultaneously (Figure 3B). Gelatin retains the key amino acid sequence, such as arginine glycine aspartate (RGD), which is critical to cell adhesion (Ahmady and Abu Samah, 2021). The proliferation of MC3T3-E1 cells was also evaluated (Figure 3C). As the cultivation time increased, the number of cells increased on both Ti2448 and Ti2448-GelMA scaffolds, without significant difference. For the Ti2448 scaffold group, the cell number at day 3 and day 5 was about 1.32 and 2.50 folds of that at day 1, respectively. For the Ti2448-GelMA scaffold group, the cell number at day 3 and day 5 was about 1.50 and 2.75 folds of that at day 1, respectively. There was no significant difference on cell numbers between these two groups at the same time point. Therefore, in our study, Ti2448-GelMA scaffold can support the cell adhesion and proliferation, demonstrating the excellent biocompatibility.

### 3.4 *In vitro* differentiation of MC3T3-E1 cells on Ti2448-GelMA scaffolds

**FIGURE 4:**
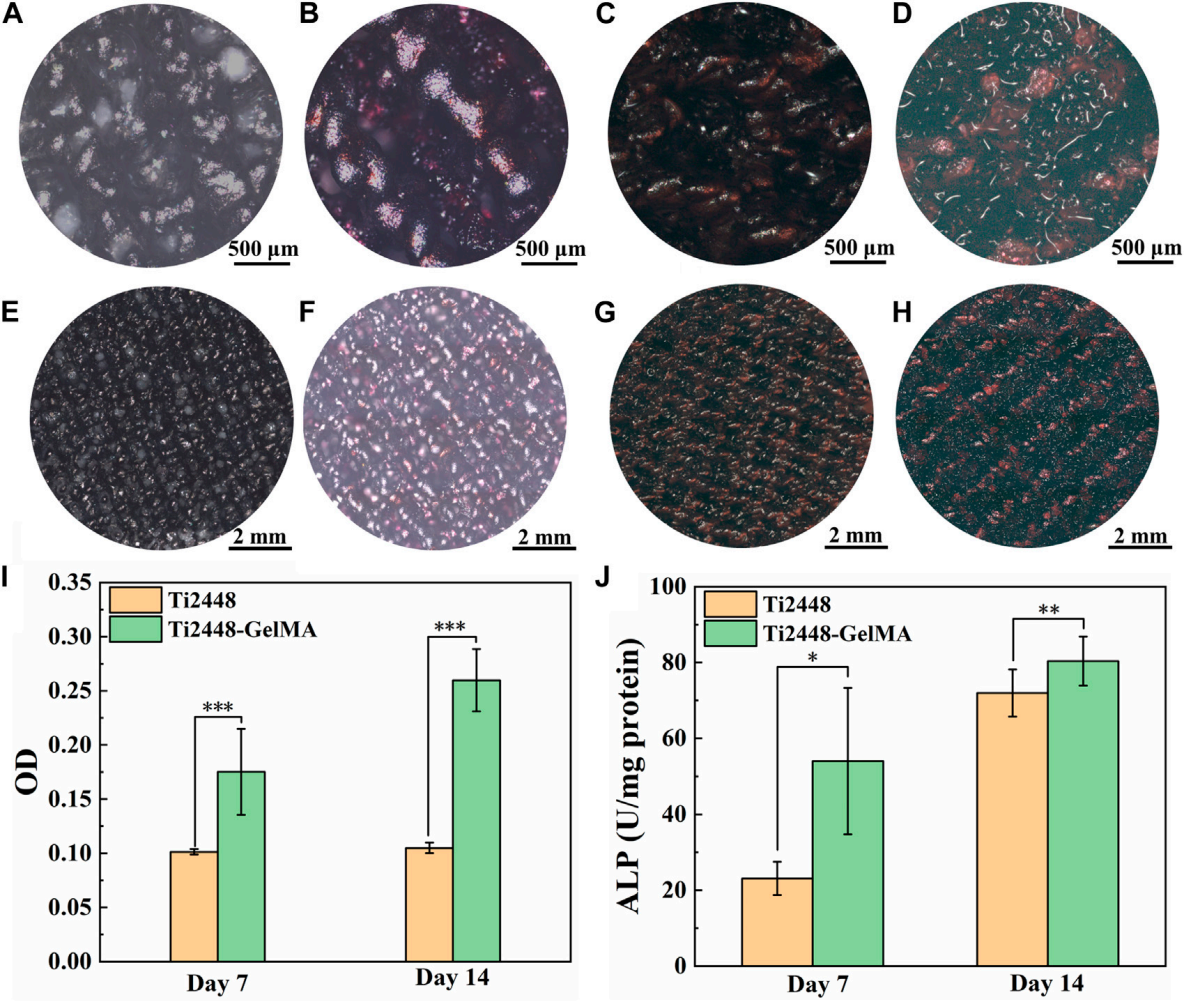
**(A–H)** The Alizarin Red S staining of MC3T3-E1 cells on **(A, E, C, G)** Ti2448, **(B, F, D, H)** Ti2448-GelMA scaffolds for **(A, B, E, F)** seven and **(C, D, G, H)** 14 days. **(I)** OD values for quantitative analysis of Alizarin Red S staining. **(J)** ALP activity of MC3T3-E1 cells cultured on different scaffolds for 7 and 14 days. Statistically significant difference is marked (*p < 0.05, **p < 0.01, ***p < 0.001).

To evaluate the osteogenic differentiation of MC3T3-E1 cells on Ti2448-GelMA scaffolds, cells were cultured for 7 and 14 days and subsequently performed ARS staining to evaluate biomineralization levels (Figures 4A–H). The results showed a relatively low degree of biomineralization for MC3T3-E1 cells cultured on the Ti2448 scaffold for 7 days, with scarcely noticeable red-stained mineralized nodules. On the other hand, the amount of mineralized nodules in the Ti2448-GelMA group was higher than the Ti2448 group after culture for 7 days. Meanwhile, the ARS staining showed progressive biomineralization with increase of culture time. After cell culture for 14 days, the Ti2448-GelMA group showed a higher amount of mineralized nodules than the Ti2448 group. Additionally, quantitative analysis showed that the amount of calcium contents from the Ti2448-GelMA group were 1.8 times and 2.5 times of that from the Ti2448 group after cell culture for 7 and 14 days, respectively (Figure 4I). Compared with the Ti2448 group, MC3T3-E1 cells in the Ti2448-GelMA group also showed significantly higher expressions of ALP activity when cultured for 7 and 14 days (Figure 4J). These results suggest that Ti2448-GelMA scaffolds can facilitate osteogenic differentiation of MC3T3-E1 cells, thereby promoting further biomineralization. GelMA recently attracts more attention, which can be used to mimic the natural bone extracellular matrix and is beneficial to vascular growth and bone regeneration (Dong et al., 2019; Heltmann-Meyer et al., 2021; Li Y et al., 2021). For example, the GelMA scaffolds were prepared by the thermally induced phase separation technique, and the ALP activity of the adipose derived stem cells cultured on the GelMA group was approximate 2 times of the untreated group after 14 and 21 days (Fang et al., 2016). In our previous work, compared with the Ti2448 scaffolds, MC3T3-E1 cells derived ECM modified Ti2448 scaffold can effectively promote osteogenic differentiation of MC3T3-E1 cells, and enhance the bone integration after implantation in rabbit femoral bone defects for 1 month (Xu et al., 2022). Therefore, the Ti2448-GelMA scaffolds were prepared by introducing the three-dimensional GelMA as the bone extracellular matrix and can be beneficial for the osteogenic differentiation of MC3T3-E1 cells.

### 3.5 Angiogenic effects of Ti2448-GelMA/DFO scaffolds

**FIGURE 5:**
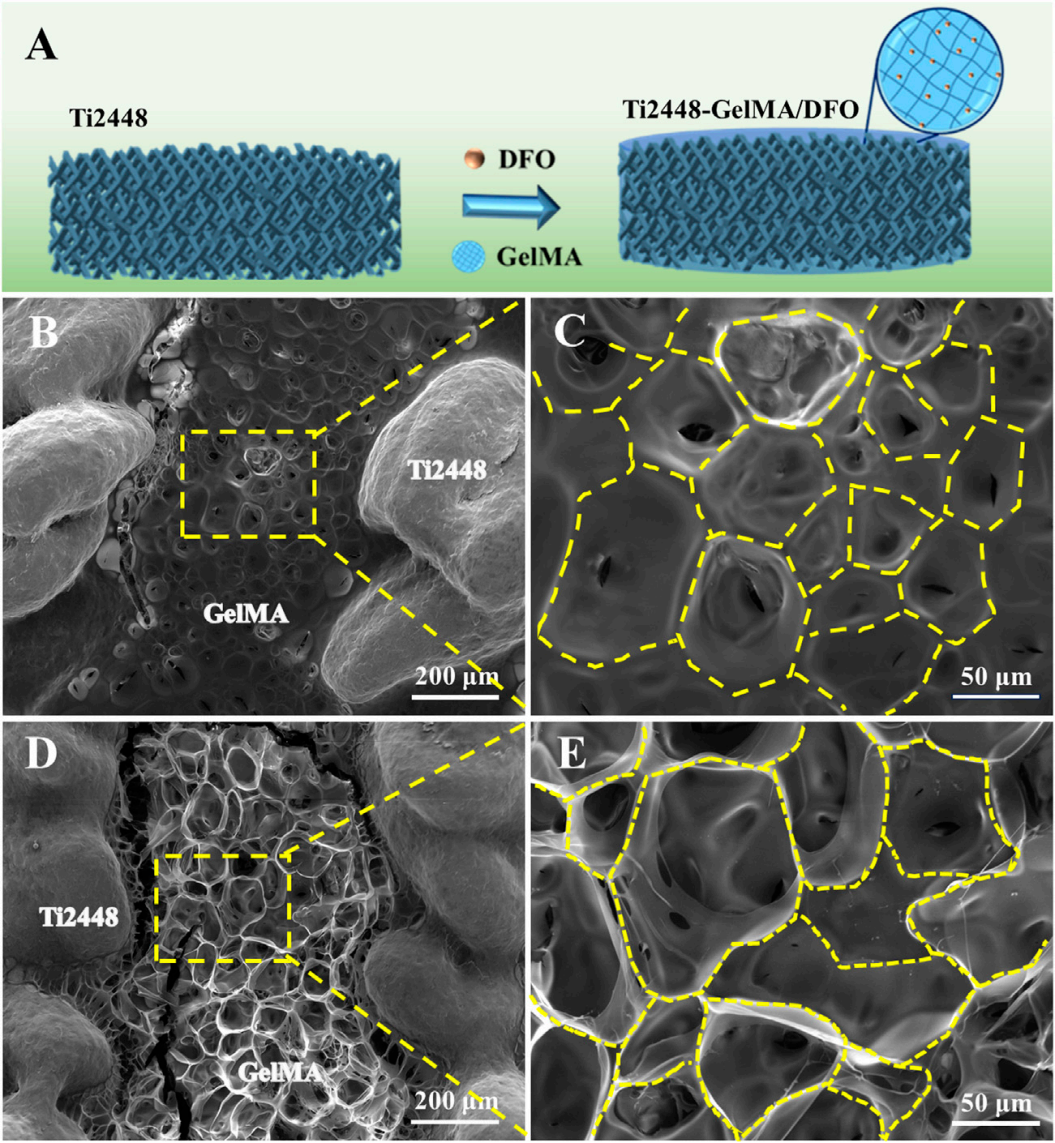
**(A)** Schematic diagram of Ti2448-GelMA/DFO scaffolds. The morphological images of **(B, C)** Ti2448-GelMA scaffold and **(D, E)** Ti2448-GelMA/DFO scaffold.

Vascularization plays an important role on the repair of large bone defects (Li et al., 2023). During bone healing and remodeling, vascular formation and bone tissue regeneration are indeed coupled processes (Ramasamy et al., 2014). Porous titanium alloys demonstrate the good osteoconductivity and osteointegration capabilities, yet the pursuit of effective internal vascularization remains a big challenge. Herein, DFO was incorporated into GelMA to enhance the Ti2448-GelMA vascularization (Figure 5A). SEM morphology revealed that doping of DFO did not change pore structures of Ti2448-GelMA/DFO scaffolds (Figures 5D, E) compared to the Ti2448-GelMA (Figures 5B, C) scaffolds.

**FIGURE 6:**
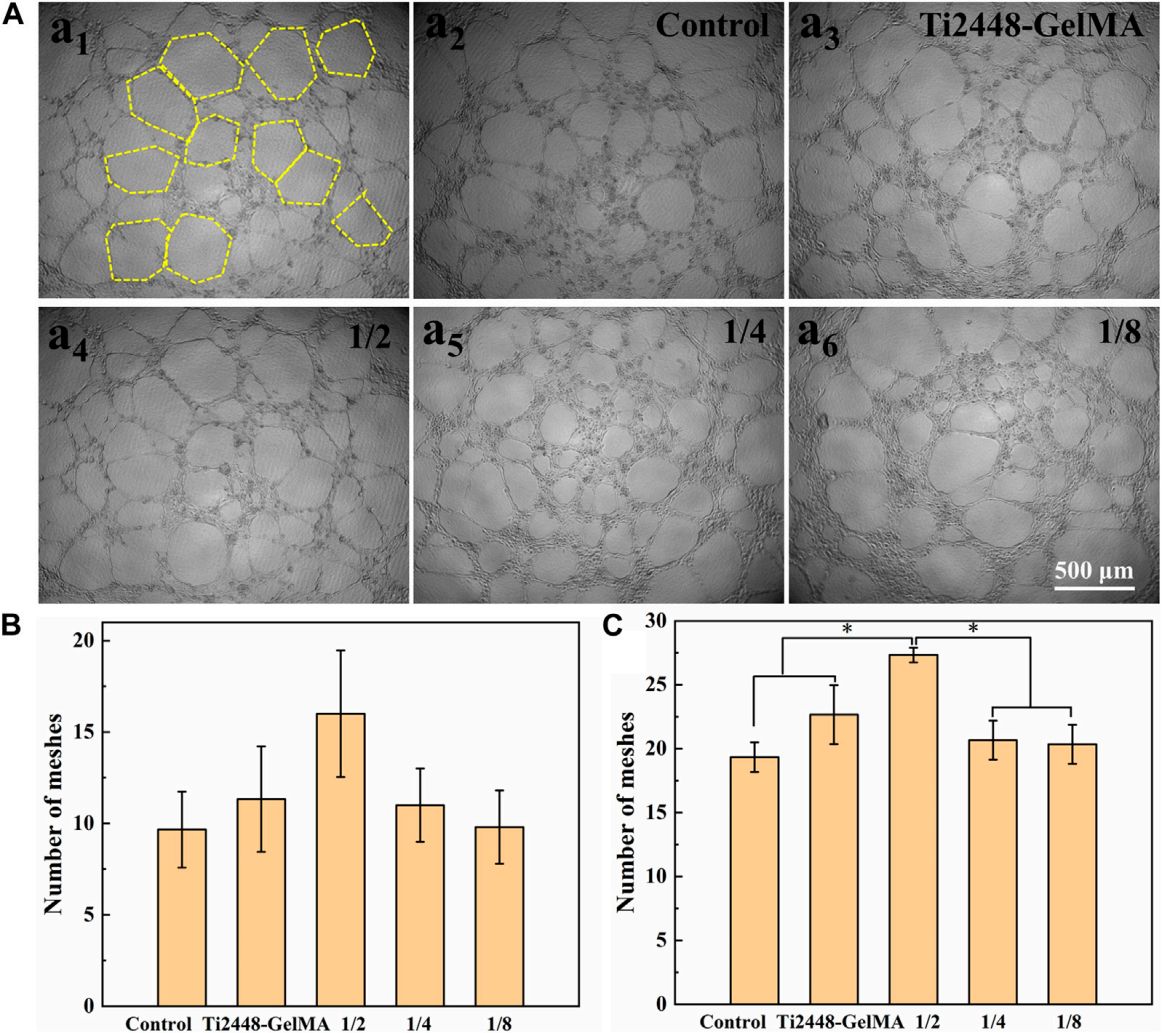
**(A)** The morphology of HUVEC cells (a_1_) on the Ti2448-GelMA/DFO extraction at 1/2 concentration for 9 h. The yellow dash lines represent the vascular meshes. The morphology of HUVEC cells on (a_2_) the control group, (a_3_) Ti2448-GelMA extraction and Ti2448-GelMA/DFO extractions at (a_4_) 1/2 concentration, (a_5_) 1/4 concentration, (a_6_) 1/8 concentration after 9 h, the number of vascular meshes at **(B)** 6 h and **(C)** 9 h. (*p < 0.05).

The angiogenic effects of Ti2448-GelMA/DFO scaffolds were evaluated by co-culture with HUVEC cells (Figure 6; Supplementary Figure S3). After cell culture for 3 h, no tubes formed on both the Ti2448-GelMA group and control group, while a small amount of tubes formed in the Ti2448-GelMA/DFO group (Supplementary Figure S3A). With increase of the cultivation time, the number and size of vascular tubes increased for all groups. After cultivation for 6 h, large tubes (∼300 μm) were observed in the Ti2448-GelMA/DFO group, compared to the Ti2448-GelMA group and control group (Supplementary Figure S3B). After culture for 9 h, a greater number of small vascular tubes consolidated into large tubes, and there were much more uniform large tubes in the Ti2448-GelMA/DFO group than the other two groups (Figure 6A). The number of vascular meshes represented the closed areas delimited by the vascular tubes marked with the yellow dash line in Figure 6A_1_. The number of vascular meshes were calculated after culture for 6 and 9 h (Figures 6B, C). The results indicated that the number of meshes for all groups increased with increase of culturing time. Additionally, the meshes increased with the increase of DFO concentration, especially for the 1/2 dilution group (7.2/mm^2^) with the higher number of vascular meshes than the other two groups (Control group: 5.3/mm^2^, Ti2448-GelMA group: 6.2/mm^2^). Therefore, HUVECs cultured with Ti2448-GelMA/DFO extractions exhibited more vascular network compared to the Ti2448-GelMA and control groups. As the concentration of DFO increases, the vascularization became more significant, demonstrating the improved vascularization effect in a dose-dependent manner. Hou et al. cultured HUVEC cells with a series of DFO concentrations (5, 20, 100 μM) for 24 h. Results showed that the group with the highest concentration of DFO (100 μM) exhibited the largest tube length and the greatest number of tubes, also demonstrating that DFO improved the tube formation of HUVECs in a concentration-dependent manner (Hou et al., 2013). Therefore, the release of DFO can effectively promote the vascularization of the Ti2448-GelMA/DFO scaffolds. Besides, the MC3T3-E1 cells attached well on the Ti2448-GelMA/DFO scaffolds (Supplementary Figure S4), which demonstrated that DFO loading on theTi2448-GelMA scaffold does not affect the cell behavior.

**FIGURE 7:**
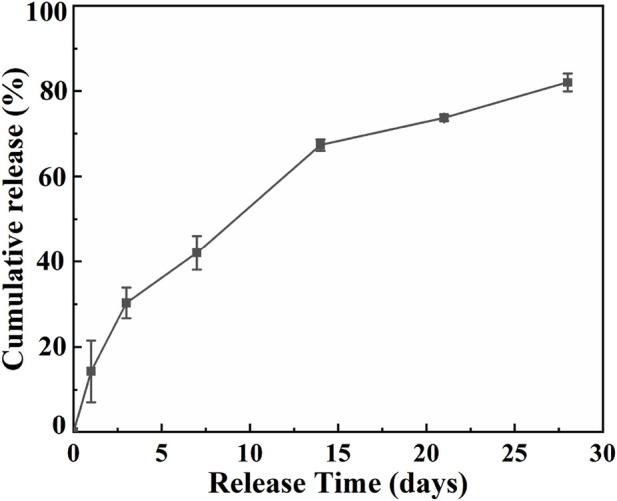
The cumulative release of DFO from the Ti2448-GelMA/DFO scaffolds.

The DFO release profile was shown in Figure 7. At the first day, DFO exhibited the release rate of nearly 14.3%, followed by a constant sustained release. After 7 days, about 42.0% DFO has been released from the Ti2448-GelMA/DFO scaffold and the final cumulative release reaches to 82.3% after 28 days. As such, Ti2448-GelMA/DFO scaffolds exhibited a sustained release of DFO possibly through the hydrogen bonding. In fact, the regeneration of blood vessels runs through the entire process of bone repair from early bone formation, maturation to remodeling (Chen et al., 2020; Simunovic and Finkenzeller, 2021). The new blood vessels infiltrated into the newly formed bone are necessitate to supply of oxygen and nutrients, as well as removal of metabolic wastes (Marenzana and Arnett, 2013). Previous research also studied the DFO release profiles *in vitro* (Cheng et al., 2018; Ran et al., 2018). For example, DFO loaded titania nanotubes substrates were prepared and then immersed into PBS solution with gently shaking at 37°C. The burst release of DFO (∼83.7%) was found in the first 12 h (Ran et al., 2018). However, immersion of the sample in a liquid solution cannot truly simulate the real release behavior of DFO from the implants *in vivo* under a mild and humid environment. Once the scaffolds are implanted in the bone defects, which will be encapsuled by the soft callus under a high humidity environment instead of immersion in a liquid solution (Salhotra et al., 2020). Thus, it is necessary to build a model mimicking the real release profile *in vivo*. Herein, Ti2448-GelMA/DFO scaffold were placed under 100% humidity at 37°C, which can well simulate the environment after implantation. The DFO release profiles in Ti2448-GelMA/DFO scaffolds can effectively achieve the sustained drug release, leading to the better vascularization for the large bone defect repair.

Composite materials have been widely studied in order to improve osteogenic activity of the scaffolds and further vascular growth for large bone defect repair (Li J et al., 2021; Qian et al., 2021; Qian et al., 2024). Herein, the Ti2448 scaffold was chosen as the main part, while GelMA was implemented as the osteogenic component, with DFO loaded as the vascularization factor. This combination will provide good mechanical properties and essential biological functions to the implant materials for large bone defect repair.

## 4 Conclusion

In this study, novel Ti2448-GelMA/DFO bone scaffolds were prepared by incorporating the GelMA loaded with DFO into porous Ti2448 scaffolds that were fabricated by the EBM method. Ti2448-GelMA scaffolds exhibited the porous network with the mean pore size of 120.2 ± 25.1 μm, and promoted the differentiation of MC3T3-E1 cells with enhanced ALP activity and mineralization. Additionally, the Ti2448-GelMA/DFO scaffolds facilitated the angiogenesis in a dose-dependent manner. Impressively, the number of vascular meshes from the Ti2448-GelMA/DFO group (7.2/mm^2^) was significantly higher than the control group (5.3/mm^2^) after cultivation of HUVECs for 9 h. The Ti2448-GelMA/DFO scaffolds also exhibited a sustained release of DFO in a humidified condition. Therefore, the Ti2448-GelMA/DFO scaffolds show great potential for large bone defect repair.

## Data Availability

The raw data supporting the conclusion of this article will be made available by the authors, without undue reservation.
