# Supplementary material for: Enhancing osteogenesis and angiogenesis functions for Ti-24Nb-4Zr-8Sn scaffolds with methacrylated gelatin and deferoxamine
Source: Front Bioeng Biotechnol. 2024 Apr 19;12:1372636. doi: 10.3389/fbioe.2024.1372636 (PMC11066197; doi:10.3389/fbioe.2024.1372636)
Supplement: Supplementary file 1 [file DataSheet1.docx]

**Supplementary Material**

**Enhancing osteogenesis and angiogenesis functions for Ti-24Nb-4Zr-8Sn scaffolds with methacrylated gelatin and deferoxamine**

Qian Xu^a,b^, Yun Bai^b,c^, Shujun Li^b,c^, Wentao Hou^b,c^, Yulin Hao^b,c^, Rui Yang^b,c^, Xiaowu Li^a,^*, Xing Zhang^b,c,^*

^a^*Department of Materials Physics and Chemistry, School of Materials Science and Engineering, Key Laboratory for Anisotropy and Texture of Materials, Ministry of Education, Northeastern University, Shenyang, Liaoning 110819, China*

^b^*Institute of Metal Research, Chinese Academy of Sciences, Shenyang, Liaoning 110016, China*

^c^*School of Materials Science and Engineering, University of Science and Technology of China, Hefei, Anhui, 230026, China*

* **Corresponding authors**:

Email address: xwli@mail.neu.edu.cn (X. Li), xingzhang@imr.ac.cn (X. Zhang)


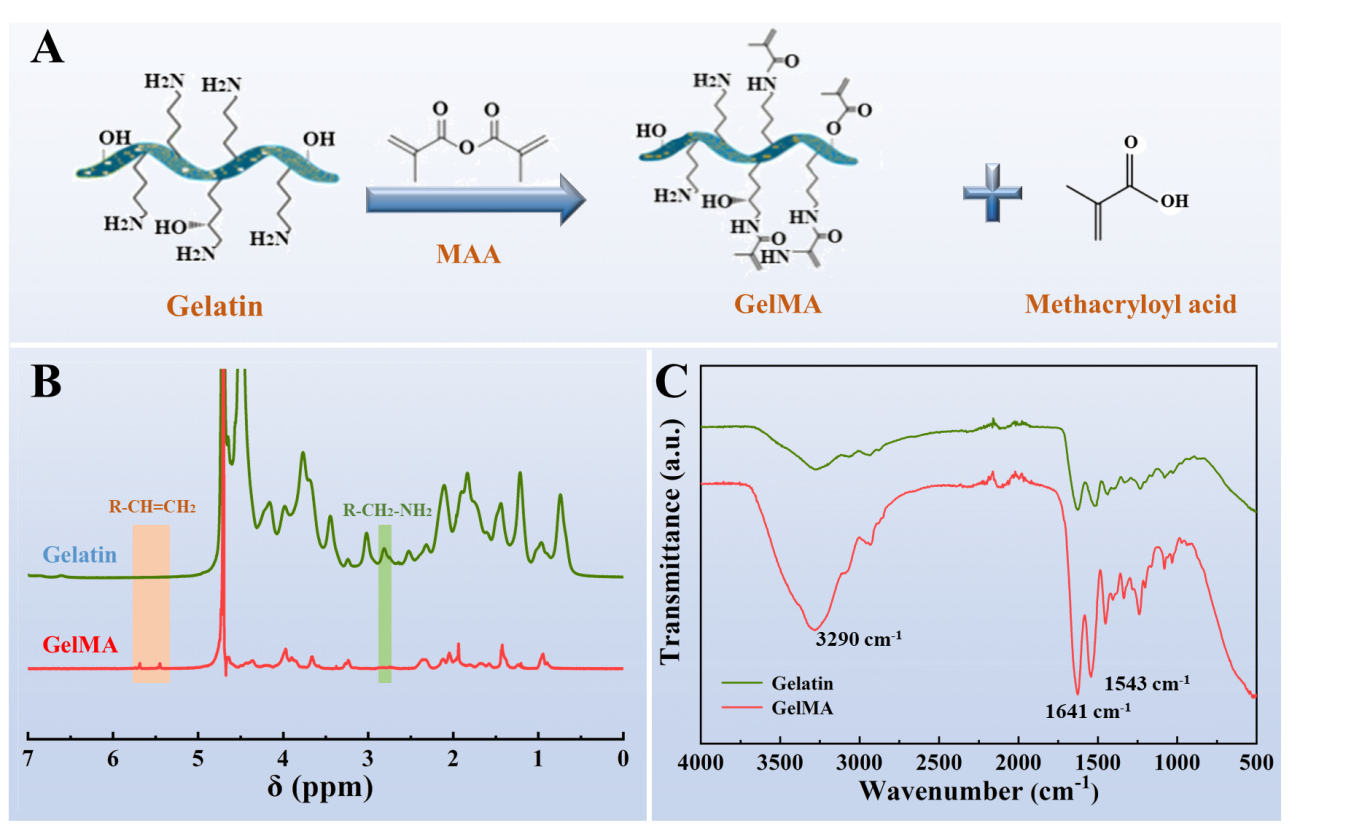


Figure S1. (A) Schematic diagram of GelMA synthesis, (B) the 1H spectra of gelatin and GelMA, (C) the FTIR spectrum of gelatin and GelMA.





Figure S2. The X-ray diffraction pattern of GelMA.


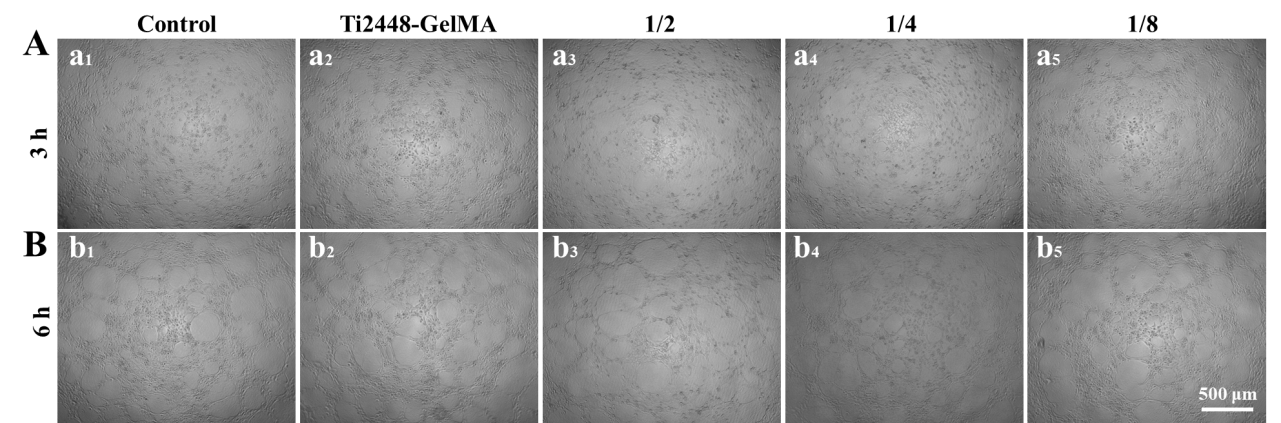


Figure S3. The morphology of HUVEC cells on (a_1_, b_1_) the control group, (a_2_, b_2_) Ti2448-GelMA extraction and Ti2448-GelMA/DFO extractions at (a_3_, b_3_) 1/2 concentration, (a_4_, b_5_) 1/4 concentration, (a_5_, b_5_) 1/8 concentration after (A) 3 hours, (B) 6 hours.


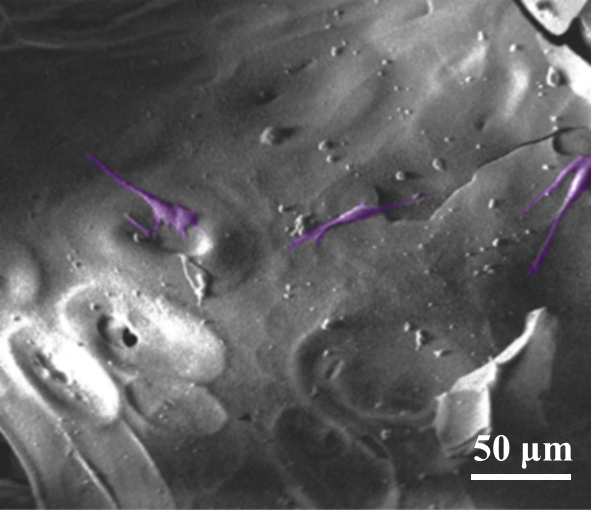


Figure S4. Cell morphology of MC3T3-E1 cells on Ti2448-GelMA/DFO scaffold for 1 day. Cells were highlighted with a pink color based on the gray values of the images.
